# Draft Genome Sequence of *Paenibacillus* sp. Strain MSt1 with Broad Antimicrobial Activity, Isolated from Malaysian Tropical Peat Swamp Soil

**DOI:** 10.1128/genomeA.01024-14

**Published:** 2014-10-09

**Authors:** Yoong Kit Aw, Kuan Shion Ong, Catherine M. Yule, Han Ming Gan, Sui Mae Lee

**Affiliations:** School of Science, Monash University Malaysia, Selangor, Malaysia

## Abstract

We report the draft genome sequence of *Paenibacillus* sp. strain MSt1, which has broad-range antimicrobial activity, isolated from tropical peat swamp soil. Genes involved in antimicrobial biosynthesis are found to be present in this genome.

## GENOME ANNOUNCEMENT

The growing number of antimicrobial-resistant bacteria (ARB) is of global concern. Infections caused by ARB mean that standard treatments no longer work, resulting in increased health care costs. Furthermore, the emergence of antibiotic resistance is not concurrent with an increase in novel antimicrobial discoveries. Only two completely new scaffolds of antibiotics have recently been found: the oxazolidinone linezolid in 2000, and the cyclic lipopeptide daptomycin in 2003 (1, 2). Therefore, there is a dire need for novel antimicrobials to combat infections caused by ARB.

*Paenibacillus* sp. strain MSt1 is a Gram-positive spore-forming rod-shaped bacterium isolated from the peat soil of the North Selangor tropical peat swamp forest, Selangor, Malaysia. *Paenibacillus* sp. MSt1 exhibits a wide range of antimicrobial activities, which includes antibacterial activities against ARB, such as methicillin-resistant *Staphylococcus aureus* (MRSA) ATCC 700699, with an MIC of the crude acetonitrile extract at 125 µg/ml. It also has antiyeast activity against *Candida albicans* (Institute for Medical Research, Malaysia) and *Cryptococcus neoformans* ATCC 66031, assayed using a spot-on assay with crude acetonitrile extract at 20 mg/ml.

The genomic DNA of a 3-day-old culture of *Paenibacillus* sp. MSt1 on tryptone soy agar (Merck, Germany) was extracted using the GF-1 DNA extraction kit (Vivantis, Malaysia) and subsequently converted into an Illumina-compatible next-generation sequencing library using Nextera XT (Illumina, San Diego, CA). The library was then sequenced on the Illumina MiSeq (150-bp paired-end reads) at the Monash University Malaysia Genomics Facility. The raw reads were trimmed and assembled *de novo* (default settings) using CLC Genomics Workbench 6 (CLC bio, Denmark). The draft genome of *Paenibacillus* sp. MSt1 has an accumulated genome size of 8,033,195 bp in 100 contigs, with an *N*_50_ of 215,818 bp. The overall G+C content was found to be 51.45%. Annotation of the genome was performed by the NCBI Prokaryotic Genome Annotation Pipeline (PGAP), and 6,455 protein-coding sequences (CDSs), 12 rRNAs, and 92 tRNAs were predicted.

Several hypothetical gene clusters that are heavily involved in the biosynthesis of antimicrobials, such as two large polyketide synthetase modules (KEQ 22505 and KEQ 22506), one large nonribosomal peptide synthetase module (KEQ 27573), and one lantibiotic-producing module (KEQ 23004) were predicted by PGAP in the genome, suggesting that *Paenibacillus* sp. MSt1 can produce more than one type of antimicrobial compound. Furthermore, although *Paenibacillus* spp. is known to produce the polymyxin class of antibiotics, the biosynthetic gene involved in the production of polymyxin was absent in the genome of *Paenibacillus* sp. MSt1. This might indicate that *Paenibacillus* sp. MSt1 produces other new antimicrobials.

Interestingly, the genome of *Paenibacillus* sp. MSt1 is currently the largest genome size known compared to other *Paenibacillus* genome sequences available (3, 4), such as that of *Paenibacillus polymyxa* (about 5.7 Mbp). The large genome size often means increased numbers of genes involved in secondary metabolites, such as antimicrobial production, are present (5).

### Nucleotide sequence accession numbers.

The draft genome sequence of *Paenibacillus* sp. MSt1 has been deposited at DDBJ/EMBL/GenBank under the accession no. JNVM00000000. The version described in this paper is the first version, JNVM00000000.1.
